# The Role of Acute Rehabilitation during the COVID-19 Pandemic: A Retrospective Study in the Czech Republic

**DOI:** 10.3390/life13051212

**Published:** 2023-05-19

**Authors:** Zdeněk Guřan, Dalibor Pastucha, Zuzana Sněhotová, Lucie Honzíková, Rastislav Maďar, Hana Tomášková

**Affiliations:** 1 Department of Rehabilitation and Sports Medicine, University Hospital of Ostrava, 708 52 Ostrava, Czech Republic; zdenek.guran@fno.cz; 2 Department of Epidemiology and Public Health Protection, Faculty of Medicine, University of Ostrava, 703 00 Ostrava, Czech Republic; 3 Department of Rehabilitation and Sports Medicine, Faculty of Medicine, University of Ostrava, 703 00 Ostrava, Czech Republic; 4 Department of Hygiene, University Hospital of Ostrava, 708 52 Ostrava, Czech Republic

**Keywords:** rehabilitation, COVID-19, physical activity, public health, physical therapy, artificial pulmonary ventilation

## Abstract

In this retrospective study, we used data from the hospital information system (HIS) to evaluate the influence of the COVID-19 pandemic on rehabilitation care at the University Hospital of Ostrava (UHO). From March 2020 to December 2021, 5173 COVID-19 cases were hospitalized at UHO. Cases within individual groups and categories are shown in a flowchart. The average patient age was 64.9 ± 16.9 years. The mean BMI value was 30.6 ± 6.8 in the rehabilitated group, which was significantly higher compared to that among the non-rehabilitated cases 29.1 ± 6.9 (*p* < 0.001). Among the admitted patients, 16.6% required artificial pulmonary ventilation (APV), 1.8% extracorporeal membrane oxygenation (ECMO), and 11.9% high-flow oxygenation (HF). The days of rehabilitation ranged from 1–102 days. Among all rehabilitated patients, 92.0% (*n* = 1302) had a hospitalization duration ranging from 1–15 days and 8.0% (*n* = 114) longer than 15 days. Overall, rehabilitation care plays an important role in providing exercise, mobilization, and rehabilitation interventions to survivors of critical illness associated with COVID-19, enabling the early and functional return to home, and it must, therefore, be integrated into the clinical care of patients with COVID-19.

## 1. Introduction

The infection known as coronavirus disease 2019 (COVID-19), reported in China in 2019, spread worldwide very rapidly and was characterized by the World Health Organization (WHO) as a pandemic on 11 March 2020. Rehabilitation units have faced the challenges of supporting both physical and cognitive recovery and have played a crucial role in reducing disability to enable the reintroduction of patients into the community. Importantly, early mobilization and rehabilitation may help to prevent or mitigate sequelae related to bed rest, thereby improving physical function and outcomes and reducing the length of hospitalization by increasing ventilator-free days [1]. At the beginning of this pandemic, general recommendations were provided based on recent information, and guidelines were developed for respiratory physiotherapy for COVID-19 patients. Pandemic restrictions have required many health facilities to limit their provision of rehabilitation services. Cancellations and limitations of planned operations and procedures have also affected the availability of rehabilitation. Some patients have avoided visits to rehabilitation clinics due to fears of infection, which has negatively affected their health [2,3,4]. 

Between 2020 and 2022, there have been many updates to the recommendations for physiotherapy management and rehabilitation among COVID-19 patients in the acute hospital setting. In our present study, we focused on the situation in the Czech Republic. The available literature mainly presents general advice. Reports from March 2020 describe the physiotherapy needs of patients in intensive care units (ICUs) who may suffer from ICU-acquired weakness or acute respiratory distress syndrome (ARDS) and recommend increased numbers of physiotherapists [5,6,7]. However, this general advice could not easily be applied. In September 2020, an update of systematic reviews related to rehabilitation and COVID-19 revealed a lack of information on this specific topic [8]. 

In April 2022, updated material regarding physiotherapy management was published. This update focused on maintaining the availability of health care, including outpatient treatment, for non-COVID-19 patients. This update stated that physiotherapy should not be routinely indicated for all patients with COVID-19 and that therapists should first use available options for indirect contact with the patient to minimize the risk of exposure to COVID-19. Moreover, a detailed examination and physiotherapy should be indicated in cases of severe disease with prolonged bed rest and critical disease [9]. 

Early recommendations for hospital-based physiotherapy were mostly defined by safety guidelines and treatment recommendations, including staffing. Treatment approaches for different phases were also established. During hospital-based physical therapy for the management of COVID-19, one should always consider the benefits of hands-on physiotherapy treatment versus the risk of virus transmission [10]. 

A rapid systematic review shows the importance of progressive exercise programs, early mobilization, and multicomponent interventions in the ICU, which can improve functional independence. The evidence regarding rehabilitation after discharge from the hospital (following an ICU admission) is inconclusive. Rehabilitation care can significantly help in shortening the length of stay at individual levels of hospitalization and substantially contributes to the improvements of patients’ functional results, such as mobility, self-sufficiency, and independence in movement and walking. There remains a need for further research to better understand the rehabilitation needs of COVID-19 patients [11].

Inpatient rehabilitation facilities should be considered as a discharge location after severe COVID-19 infections. The average length of stay at an inpatient rehabilitation facility is reportedly about 11 days, although it is unclear what percentage of patients received inpatient rehabilitation [12]. One study has reported a good experience with the creation of a specialized rehabilitation hospital for post-acute conditions of COVID-19 [13]. It seems that among all patients hospitalized for COVID-19, about 25% require rehabilitation for post-COVID-19 conditions [14], with the greatest post-acute rehabilitation needs being among patients with co-morbidities, such as hypertension, diabetes, or coronary diseases [15]. 

One study reported the inpatient bed occupancy and the predicted need for rehabilitation in two large teaching hospitals in London. It captured data from just a single day to predict the rehabilitation needs after discharge and established a model, including four pathways of discharge. The study predicted that 50% of people would be simply discharged without further need for care, ~45% would be able to return home with some kind of support from health or social care, ~4% might need rehabilitation in a bedrest setting, and the remaining 1% would not be able to be discharged from acute care. The authors collected real data and compared them with this predicted model. They focused on the last two pathways (i.e., the last 5% of patients), and they found that the real percentages of patients following these two discharge pathways exceeded those expected in the assessed model by more than five times [16]. Several factors have been identified as potentially associated with post-acute rehabilitation needs, including age above 65 years, oxygen needs, status after mechanical ventilation or tracheostomy, dyspnea, and a high level of dependency for activities of daily living [17,18,19].

The clinical characteristics of post-ICU patients often include exercise-induced desaturation (an arterial oxygen saturation lower than 88%), muscle weakness, and a low outcome in functional tests [20]. To identify the benefits of long-term rehabilitation, it is recommended to use variable measurement tools for objectivization. Most studies have focused on functional outcomes and have used a tool like the Barthel index, 10 sit-to-stand test, or grip strength test [20,21,22]. 

Risk factors for hospital admission have been set, as described above, and about 6% were admitted to the ICU only and 11% were re-admitted after discharge [23,24,25]. Objective tools for discharge have been set, especially in terms of respiratory values [26,27,28]. 

Health workers may also suffer from post-acute COVID-19 syndrome, with some persisting symptoms, such as fatigue, shortness of breath, etc. [29]. Respiratory and musculoskeletal or neuropsychological conditions after four weeks might be identified as long COVID-19 signs [30]. Some data show discrepancies between the estimated importance of specific examinations and treatment techniques and the level of current experience among physiotherapists. Such findings indicate an urgent need to develop new professional education and training programs with a focus on the interdisciplinary rehabilitation of patients with post-COVID-19 syndrome [31,32].

Our research questions were as follows: “What is the most important factor associated with the need for, and length of, rehabilitation required during acute hospitalization?” and “What are the actual caseloads in different discharge categories?”.

## 2. Materials and Methods

### 2.1. Data Settings

This retrospective study from the hospital information system (HIS) was approved by the Local Ethics Committee, University Hospital Ostrava nr. 971/2021. We evaluated data from the period of March 2020–December 2021. The analysis included only patients 18–99 years of age and did not include any data from the pediatric population. We also excluded data from patients with psychiatric diagnoses or patients in palliative care. The analyzed data set included repeated hospitalizations of the same person, including a new hospitalization in a different department of the hospital (transfer to another department). This inclusion is important in terms of the number of referrals for rehabilitation for the same patient. Cases within individual groups and categories are shown in a flowchart (Supplement S1). 

Our results mainly focused on the topics of intensive care and BMI. We identified groups of cases admitted to intensive care units, including those receiving artificial pulmonary ventilation (APV), extracorporeal membrane oxygenation (ECMO), or high-flow oxygenation (HF). We also identified cases of patients with obesity (BMI > 30). We compared the cases that underwent some kind of rehabilitation treatment (rehab group) versus those that were not indicated for rehabilitation at all (non-rehab group). Multiple comparisons were made among these groups.

Among the hospitalized patients, we identified the number of health transfers to different types of care, discharges to home, and the number of deaths. We particularly focused on the discharge of the rehabilitation group. The cases can be divided into four general categories: Category A, cases discharged to home or outpatient care; Category B, cases requiring follow-up hospitalization in an acute-care bed of the same or another medical facility; Category C, cases requiring follow-up hospitalization in a follow-up-care bed of the same or another medical facility or requiring transfer to a social care facility; and Category D, cases of death.

### 2.2. Statistical Methods

Data regarding the quantitative parameters were processed by descriptive statistics using the mean and standard deviation. Qualitative data were presented as the number of observations (*n*, %). No imputations were performed for missing data. Patients with missing body mass index (BMI) data were excluded from the statistical analysis for the relevant parameters. Differences in proportions were examined using a chi-squared test. Before the statistical testing of quantitative parameters, the normality of the data was verified using the Shapiro–Wilk test. If normality was rejected, non-parametric methods were used. Between-group comparisons were made using the independent sample *t*-test and F-test in the case of normally distributed data and non-parametric Mann–Whitney U test. Correlations between different types of scores were explored by the Spearman and Pearson correlation coefficient. All tests were performed at a 5% significance level. Statistical analyses were performed using the software Stata/BE 17.0.

## 3. Results

### 3.1. Basic Characteristics of the Study Group

There was a total of 5173 cases of COVID-19 admitted to the University Hospital Ostrava during March 2020–December 2021. The average age was 64.9 ± 16.9; males constituted 54% (2783/5173) and females 46% (2390/5173) of cases. There were 27.4% (1416) of cases in the rehabilitation group. 

**Figure 1:**
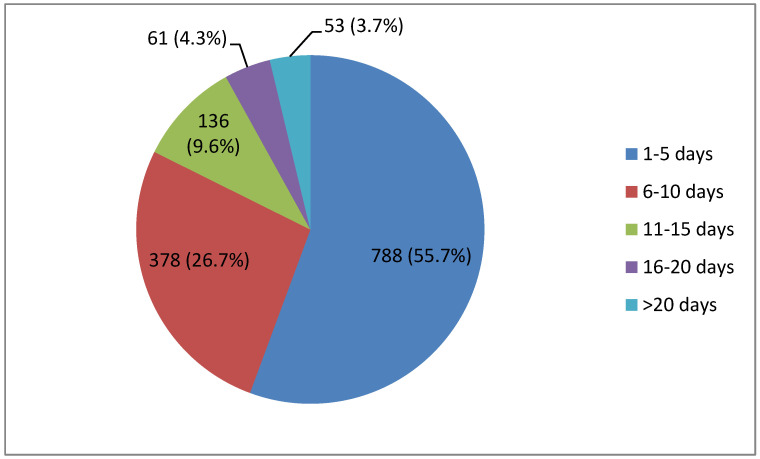
Duration of rehabilitation (*n* = 1416).

The duration of active rehabilitation ranged from 1–102 days. Within the rehab group, 92.0% (1302/1416) had a rehabilitation duration ranging from 1–15 days, while the remaining 8.0% (114/1416) underwent rehabilitation for longer than 15 days (Figure 1).

**Figure 2:**
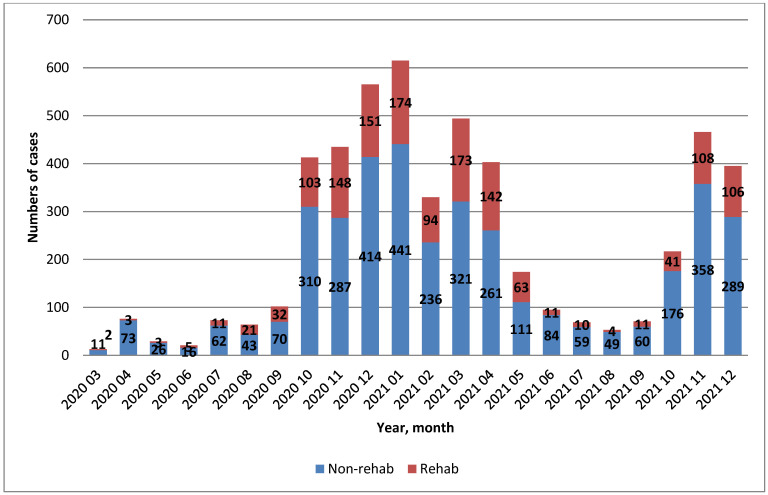
Number of cases in the rehabilitated and non-rehabilitated groups during each month of the study period (*n* = 5173).

Figure 2 shows comparisons of the rehab and non-rehab groups during the whole study period.

Rehabilitation was indicated in 27.4% (1416/5173) of cases, which included 31% of male patients (850/2783) and 24% of female patients (566/2390). The mean age of the rehabilitated patients was 64.6 years, not significantly different from the non-rehabilitated groups. A detailed description of the groups is shown in Table 1.

### 3.2. BMI and Cases from Intensive Care

We particularly focused on the cases that required ICU admission for APV, ECMO, or HF, as well as cases of patients with obesity. The analysis of BMI included 4770 cases due to missing data. Within this sample, 40% of patients had obesity (BMI > 30). The mean BMI value was 30.6 ± 6.8 in the rehabilitated group, which was significantly higher compared to that among the non-rehabilitated cases of 29.1 ± 6.9 (*p* < 0.001) (Table 1).

Among all analyzed hospitalizations, 16.6% involved APV, 1.8% ECMO, and 11.9% HF. Not all of the patients requiring these treatments were indicated for rehabilitation.

The rehab and non-rehab groups significantly differed in their rates of APV (40% vs. 8%; *p* < 0.001), ECMO (5% vs. 0.6%; *p* < 0.001), and HF (22% vs. 8%; *p* < 0.001) (Table 1).

Among patients who required APV, ECMO or HF, the BMI value was significantly higher in the rehab group compared to the non-rehab group (*p* = 0.002) (Table 1 and Table 2).

### 3.3. Evaluation of the Difficulty of Rehabilitation

Among patients who underwent rehabilitation, we monitored the need to delegate more than one therapist to the intervention. Assistance (i.e., more than one therapist) was required for 337 cases (23.8% of the rehab group and 6.5% of the entire set). After excluding cases from this group that were missing BMI data, the average BMI value was 30.1 ± 6.6 among the 323 cases that required more than one therapist for rehabilitation. The distribution of BMI did not significantly differ according to the need for assistance in rehabilitation (*p* = 0.454) (Table 2).

### 3.4. Termination of Hospitalization

**Figure 3:**
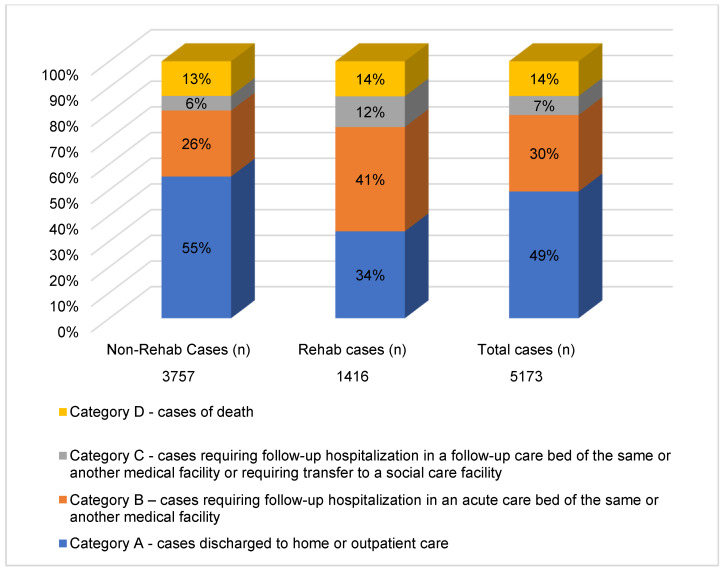
Termination of hospitalization in the rehab vs. non-rehab groups (*n* = 1416/3757).

Of the total number of patients hospitalized with COVID-19, 14% died (group D), 37% were transferred to different types of care (groups B and C), and 41% were discharged home (group A) (Figure 3).

Compared to the non-rehab group, the rehab group exhibited significantly worse outcomes in terms of the category of discharge (*p* < 0.001). The percentages of patients discharged home were 34% in the rehab group vs. 55% in the non-rehab group. The corresponding percentages of patients requiring subsequent hospitalization (categories B + C) were 52% vs. 32%, respectively, and the corresponding percentages of patients who died were 14% in the rehab group and 13% in the non-rehab group (Figure 3).

## 4. Discussion

In this study, we evaluated the established procedures using available data from the regional university hospital (a region with a population of approximately 1.2 million inhabitants). The aim was to outline an ideal model of the rehabilitation process and adequate rehabilitation capacity. Our findings and general recommendations for rehabilitation in the ICU have been incorporated into the care of our patients. 

Early and daily mobilization is applied 5–6 times/week, including at least a 30 min exercise session as part of a progressive exercise program [11]. For patients with COVID-19, their current state of health and ability to handle the exercise load is decisive regarding their length of rehabilitation. Notably, the need for early rehabilitation applies only to a certain percentage of high-risk patients who are admitted to the ICU. Rehabilitation is required for up to 67% of patients on APV, 78% of patients on ECMO, and ~50% of patients on HF. Personnel capacities in intensive care had to be substantially strengthened. Some authors have reported that physiotherapy management in these acute wards is focused on three aspects: respiratory, motor, and prevention of complications [25].

The follow-up acute rehabilitation program was focused on patients who no longer had to be in the intensive care unit, whose condition (due to prolonged hospitalization or severe course of COVID-19) required further rehabilitation and who could not be discharged to home treatment due to their current state of health. Among patients who received the rehabilitation intervention in the acute and sub-acute departments, 92% of rehabilitation interventions lasted between 1–15 days. The rehabilitation intervention lasted longer than 15 days in only a small percentage of patients (8%), which corresponds to the conclusions of other authors [12].

Although we did not establish a rehabilitation hospital [13], parts of some workplaces were set aside to deal with patients who had experienced COVID-19 and the need for rehabilitation. The planning of these capacities and their subsequent use have not been evaluated in the Czech Republic and are not the subject of the study. Our healthcare system did not choose to establish specific hospitals for rehabilitation after COVID-19, however, rehabilitation capacities were allocated across the healthcare system in the Czech Republic.

At the beginning of the COVID-19 pandemic, there was uncertainty about the indications for physiotherapy. This hesitation is why the number of indicated cases was low during the first two months. In the following months, the number of cases increased (roughly from May 2020) because it became necessary to focus on cases where rehabilitation was clearly indicated. Rehabilitation was needed for cases with a prolonged course of the disease, and effort was made to ensure that rehabilitation could be provided when required. Although we had already perceived the need for rehabilitation in the initial stages of the pandemic, this need had to be addressed with respect for the maximum safety of medical personnel. There was a certain lack of personal protective equipment (PPE) available for the field of rehabilitation, as priority was given to nursing and intensive care.

The availability of further follow-up rehabilitation should also depend on the patient’s limitations in functions, activities, and participation, as defined by the International Classification of Functioning, Disability and Health (ICF). We presently monitor the severity of disability only for hospitalizations in acute rehabilitation, not in detail for patients in the ICU. The current system only uses the Barthel index assessment, which we consider insufficient. This tool was not used as a standard upon discharge from the ICU.

Regarding follow-up outpatient care options, there is currently only screening in the so-called post-COVID-19 outpatient clinic for patients who have experienced COVID-19. Patients are not targeted. Post-ICU follow-up clinics are also available, which provide more systematic monitoring. In this area, we see the current system of recording severe cases of COVID-19, including a more detailed assessment of each patient’s functional abilities.

A number of studies have tried to define the relationship between obesity and COVID-19 severity or death. Moreover, several other conditions have been examined in this context, including asthma, chronic kidney disease, and diabetes. Our present analysis revealed that the BMI was significantly higher in the group that underwent rehabilitation. We also found that each of the groups that received APV, ECMO, or HF had a higher need for rehabilitation. Other studies have reported an association between hospitalization and obesity [26,27]. The reported duration of stay in the ICU has ranged from 5–19 days and the subsequent overall rehabilitation length of stay from 10–14 days. Analysis of health system issues has also identified the lack of an existing comprehensive rehabilitation system or a disaster-response system that includes rehabilitation. Data from a 1.5-month period at a teaching hospital in France supports the observation that ICU-acquired weakness (estimated in 88% of patients in the ICU) and difficulty in weaning patients from APV (9%) may be associated with post-ICU rehabilitation needs [17,18,19].

The rehabilitation process for COVID-19 patients has higher time requirements. This includes the time that must be devoted to preparation before and after rehabilitation (management of hygiene and protective work equipment), as well as a longer time for the management of the patient in isolation mode and a longer rehabilitation process itself. In patients with a severe and critical disease course, we can expect that physiotherapy will not be well-managed by just one therapist, even in cases of assistance within the framework of a multidisciplinary team. Particularly in cases where a patient has a higher risk profile (e.g., in terms of age, BMI, comorbidities, APV-dependence, etc.), it can be assumed that the assistance of another skilled physiotherapist will be necessary, for example, to help with patient verticalization and mobilization. These factors increase the number of physiotherapists needed to provide healthcare for these patients. Physiotherapy was profiled, particularly in cases with a severe and critical course of the disease and in patients requiring a stay in the ICU. According to the currently available data, the next group of patients requiring physiotherapy will be the patients with post-COVID-19 syndrome.

We identified the need to define and then evaluate the necessity of involving another therapist in the rehabilitation process, with the mean BMI as well as the receipt of APV, ECMO, or HF as factors. It turns out that a high BMI value was not statistically significant and, therefore, the only determining factor for the need for a second therapist. These parameters were not previously monitored, or it was not possible to read them from the code designations of the reported rehabilitation. That is why the so-called signal code was introduced to help another therapist. The implementation of the code within therapy helped on two fundamental levels. The first level is the forensic point of view, which enables us to clearly identify another member of the team who worked with the patient. The second level is the possibility of retrospectively determining whether the given patient had demanding needs from the perspective of rehabilitation management. This system enables better monitoring of the necessity for involvement and participation of the individual team members.

Based on the current limited evidence, disease management of long-COVID-19 signs and symptoms will require a holistic longitudinal follow-up in primary care, multidisciplinary rehabilitation services, and the empowerment of affected patient groups [27,28,29,30,31]. The use of remote telerehabilitation elements may be helpful. The goal is to provide rehabilitation where it is absolutely necessary and in a manner that is safe and effective, both in the field of acute care and also in the aftermath of COVID-19 illness. At the same time, it is also necessary to maintain sufficient capacities of acute rehabilitation for other rehabilitation diagnoses (e.g., strokes, polytraumas, etc.).

This study may have several limitations. The study covers a relatively long period of the COVID-19 pandemic in the Czech Republic. During this time period, there were mutations of the virus and gradually increasing vaccination coverage among the population. These changes could have significantly affected the course and symptomatology of the disease and thereby also affected the need for rehabilitation. Additionally, the BMI was not detected in all patients, especially in patients who were admitted in a critical condition. At the same time, it was not possible to validly verify data from other parts of the patient’s documentation. The available data were not up-to-date due to recent hospitalization.

Identification of this item in the patient record also appears to be a problem. The entry in the documentation was optional and not subject to automatic error checking. We recommend this as a mandatory field with a check of the entered data.

## 5. Conclusions

Our analysis showed that the studied university hospital and its available rehabilitation care, including acute and subsequent rehabilitation beds, provide sufficient facilities for the treatment of a severe course of COVID-19 and for the rehabilitation treatment of conditions associated with COVID-19. Ensuring rehabilitation in an acute intensive care unit required a significant modification of regular programs and the increased availability of staff to meet the needs of newly emerging units for patients with COVID-19. In ensuring an adequate regimen in terms of hygiene and protective work equipment, we noticed difficulties only in terms of the initial availability of suitable PPEs and the insufficient information about the disease at the beginning of the pandemic.

Additionally, acute rehabilitation services were less accessible to patients in non-COVID-19 diagnostic groups. Further analysis will be required to examine the comparative data on accessibility for stroke patients. The COVID-19 pandemic has revealed the vulnerabilities of healthcare systems and the importance of ensuring the availability of quality care for all patients. 

Governments and healthcare institutions must focus on minimizing the impact of the pandemic on rehabilitation, making efforts to strengthen and protect health systems, and creating support programs to help cope with the impact of the pandemic on rehabilitation. Such efforts may include financing new technologies for online rehabilitation, increasing the capacity of rehabilitation centers, ensuring sufficient supplies of protective equipment, and training a sufficient number of personnel. The recommendation is also towards better equipment in critical care, e.g., beds with automatic measurement of the patient’s weight, automatic reading and transfer to the information system and patient documentation, etc. Greater emphasis should also be placed on staff training in working with specific personal protective equipment, as this will also improve work efficiency and speed up the care of infectious patients.

Rehabilitation units do not have enough isolation rooms available. This results in a limitation and a reduction in the available capacity of beds for the needs of rehabilitation. This should be a part of planning, for example, during building renovations.

## Figures and Tables

**Table 1:** Detailed rates in the rehab and non-rehab groups.

Groups	Non-Rehab *n* (%)	Rehab *n* (%)	Total *n* (%)	*p*-Value (*t*-Test/Chi^2^ Test)
Males *n* (%)	1933 (51.5)	850 (60.0)	2783 (53.8)	
Females *n* (%)	1824 (48.5)	566 (39.9)	2390 (46.2)	
Total *n* (%)	3757 (72.6)	1416 (27.4)	5173 (100)	*p* < 0.001 ^2^
Mean age	65.0	64.6	64.9	*p* = 0.49
Age Std. dev.	17.6	14.6	16.9	
BMI < 24.99 *n* (%)	980 (28.5)	264 (19.8)	1244 (26.0)	
BMI 25–29.99 *n* (%)	1193 (34.7)	431 (32.3)	1624 (34.0)	
BMI 30–34.99 *n* (%)	770 (22.4)	353 (26.4)	1123 (23.5)	
BMI > 35 *n* (%)	500 (14.5)	288 (21.6)	788 (16.5)	
BMI Total ^1^ *n* (%)	3443 (72.0)	1336 (28.0)	4779 (100)	*p* < 0.001 ^3^
Mean BMI	29.1	30.6	29.5	*p* < 0.001
BMI Std. Dev.	6.9	6.8	6.9	
Non-APV *n* (%)	3472 (92.4)	844 (59.6)	4316 (83.4)	
APV *n* (%)	285 (7.6)	572 (40.4)	857 (16.6)	*p* <0.001 ^3^
Non-ECMO *n* (%)	3736 (99.4)	1346 (95.1)	5082 (98.2)	
ECMO *n* (%)	21 (0.6)	70 (4.9)	91 (1.8)	*p* < 0.001
Non-HF *n* (%)	3450 (91.8)	1105 (78.0)	4555 (88.0)	
HF *n* (%)	307 (8.2)	311 (22.0)	618 (12.0)	*p* < 0.001

^1^ Missing data were excluded. ^2^ *t*-test. ^3^ chi^2^ test.

**Table 2:** BMI distribution according to the different group.

Groups	BMI < 24.99 *n* (%)	BMI 25–29.99 *n* (%)	BMI 30–34.99 *n* (%)	BMI > 35 *n* (%)	BMI Total ^1^ *n* (%)	*p*-Value (Chi^2^ Test)
Non-APV *n* (%)	1144 (28.9)	1374 (34.8)	884 (22.4)	552 (14.0)	3954 (100)	
APV *n* (%)	100 (12.1)	250 (30.3)	239 (29.0)	236 (28.6)	825 (100)	*p* < 0.001
Non-ECMO *n* (%)	1237 (26.4)	1605 (34.2)	1095 (23.4)	753 (16.1)	4690 (100)	
ECMO *n*(%)	7 (7.9)	19 (21.4)	28 (31.5)	35 (39.3)	89 (100)	*p* < 0.001
Non-HF *n* (%)	1153 (27.6)	1432 (34.2)	959(22.9)	641 (15.3)	4185 (100)	
HF *n* (%)	91 (15.3)	192 (32.3)	164 (27.6)	147 (24.8)	594 (100)	*p* < 0.001
Total BMI group *n* (%)	1244 (26.0)	1624 (34.0)	1123 (23.5)	788 (16.5)	4779 (100)	
Single therapist *n* (%)	195 (19.3)	320 (31.6)	277 (27.4)	221 (21.8)	1013 (100)	
Second assistant *n* (%)	69 (21.4)	111 (34.4)	76 (23.5)	67 (20.7)	323 (100)	*p* = 0.454
Total Single and Assistant group *n* (%)	264 (19.8)	431 (32.3)	353 (26.3)	288 (21.6)	1336 (100)	

^1^ Cases with missing BMI data were excluded, such that this analysis included only 323 of 337 cases for which a second therapist was required for rehabilitation.

## Data Availability

The data presented in this study are available upon request from the corresponding author. The data are not publicly available due to ethical restrictions and the privacy of the patients.

## Supplementary Materials

The following supporting information can be downloaded at: https://www.mdpi.com/article/10.3390/life13051212/s1, Figure S1: Cases in individual groups and categories; Table S1: STROBE statement—checklist.
