# Supplementary material for: The Role of Acute Rehabilitation during the COVID-19 Pandemic: A Retrospective Study in the Czech Republic
Source: Life (Basel). 2023 May 19;13(5):1212. doi: 10.3390/life13051212 (PMC10221685; doi:10.3390/life13051212)
Supplement: Supplementary file 1 [file life-13-01212-s001.zip › life-2356914-supplementary.pdf]

Supplement S1

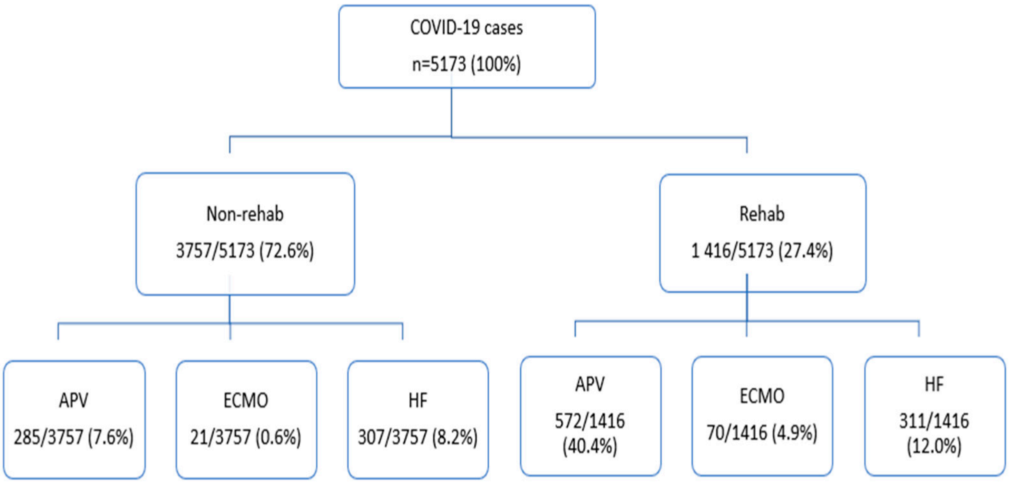

Figure S1. Cases in individual groups and categories

	Item No.	Recommendation	Page No.	Relevant text from manuscript
**Title and abstract**	1	(*a*) Indicate the study's design with a commonly used term in the title or the abstract	1	The Role of Acute Rehabilitation during the COVID-19 Pandemic: A Retrospective Study in the Czech Republic
		(*b*) Provide in the abstract an informative and balanced summary of what was done and what was found	1	
**Introduction**				
Background/rationale	2	Explain the scientific background and rationale for the investigation being reported	2-3	
Objectives	3	State specific objectives, including any prespecified hypotheses	3	
**Methods**				
Study design	4	Present key elements of study design early in the paper	3-4	retrospective study
Setting	5	Describe the setting, locations, and relevant dates, including periods of recruitment, exposure, follow-up, and data collection	3-4	the hospital information system
Participants	6	Cohort	3-4	COVID-19 patients 18–99 years of age
		Cases and controls	3-4	rehab group versus non-rehab group

Variables	7	Clearly define all outcomes, exposures, predictors, potential confounders, and effect modifiers. Give diagnostic criteria, if applicable	3-4	Gender, age, BMI, length of hospitalization, termination of hospitalization
Data sources/ measurement	8*	For each variable of interest, give sources of data and details of methods of assessment (measurement). Describe comparability of assessment methods if there is more than one group	3-4	Sources - the hospital information system
Bias	9	Describe any efforts to address potential sources of bias		COVID-19 patients, who were hospitalized in University Hospital Ostrava
Study size	10	Explain how the study size was arrived at	3-4	All COVID-19 patients from University Hospital Ostrava during the period March 2020–December 2021.
Quantitative variables	11	Explain how quantitative variables were handled in the analyses. If applicable, describe which groupings were chosen and why	4	Before statistical testing of quantitative parameters, the normality of the data was verified using the Shapiro-Wilk test.
Statistical methods	12	Describe any methods used to examine subgroups and interactions	4	Non-parametric test
		Explain how missing data were addressed	4	Patients with missing body mass index (BMI) data were excluded from the statistical

				analysis for the relevant parameters
**Results**				
Participants	13	(a) Report numbers of individuals at each stage of study—eg numbers potentially eligible, examined for eligibility, confirmed eligible, included in the study, completing follow-up, and analysed	4	*n* = 5173, rehab 1416, non-rehab 3757
		(b) Give reasons for non-participation at each stage		There are any non-participations
		(c) Consider use of a flow diagram		Supplement 1
Descriptive data	14	(a) Give characteristics of study participants (eg demographic, clinical, social) and information on exposures and potential confounders	4	3.1. Basic Characteristics of the Study Group
		(b) Indicate number of participants with missing data for each variable of interest	7	Table 2 – missing BMI
Outcome data	15	*Cohort study*—Report numbers of outcome events or summary measures over time	4-5	3.1. Basic Characteristics of the Study Group, Fig. 1, Fig. 2
		Case-control study—Report numbers in each exposure	6	Table 1

		category, or summary measures of exposure		
Main results	16	Give unadjusted estimates and, if applicable, confounder-adjusted estimates and their precision (eg, 95% confidence interval). Make clear which confounders were adjusted for and why they were included	6-8	3.3. Evaluation of the Difficulty of Rehabilitation, Tab. 2<br>3.4. Termination of hospitalization
Other analyses	17	Report other analyses done—eg analyses of subgroups and interactions, and sensitivity analyses		No
**Discussion**				
Key results	18	Summarise key results with reference to study objectives	8-10	
Limitations	19	Discuss limitations of the study, taking into account sources of potential bias or imprecision. Discuss both direction and magnitude of any potential bias	8-10	
Interpretation	20	Give a cautious overall interpretation of results considering objectives, limitations, multiplicity of analyses, results from similar studies, and other relevant evidence	8-10	

Generalisability	21	Discuss the generalisability (external validity) of the study results	8-10	
**Other information**				
Funding	22	Give the source of funding and the role of the funders for the present study and, if applicable, for the original study on which the present article is based	11	This research was supported by the Ministry of Health of the Czech Republic, grant no. NU22-A-114.

Table S1. STROBE statement - checklist
